# Prévalence du tabagisme chez le personnel médical et paramédical du CHU Mohamed VI à Marrakech

**DOI:** 10.11604/pamj.2017.26.45.10872

**Published:** 2017-01-31

**Authors:** Farid Badri, Hafsa Sajiai, Lamyae Amro

**Affiliations:** 1 Service de Pneumologie, Hôpital Arrazi, CHU Mohammed VI, Marrakech, Maroc

**Keywords:** Tabagisme, hôpital, sevrage, Smoking, hospital, withdrawal

## Abstract

Le tabagisme est un problème majeur de santé publique. Le personnel médical et paramédical n'est pas épargné par ce fléau. L'interdiction de fumer à l'hôpital est née d'une volonté gouvernementale de réduire le tabagisme passif. L'objectif de cette étude était d'évaluer les habitudes tabagiques chez le personnel soignant médical et paramédical, afin d'élaborer une stratégie de lutte contre le tabagisme au sein de cette population, et de les adresser éventuellement à la consultation d'aide au sevrage tabagique. L'étude transversale descriptive concernant l'ensemble du personnel soignant du CHU de Marrakech en se basant sur la distribution des questionnaires anonymes. Nous avons distribué 530 questionnaires dont 380 ont été récupérés, soit un taux de réponse de 71,7%. La population d'étude était constituée de 58,2% de femmes (n= 221) et de 41,8% d'hommes (n=159). Le personnel médical (n=220) était la catégorie professionnelle la plus représentée (57,9%) suivie des infirmiers (31,8%). Les fumeurs (n = 62) représentaient 16,3% de notre population d'étude; les ex-fumeurs (n=31) 8,1% et les non-fumeurs (n= 287) 75,5%. La moyenne d'âge des fumeurs était de 31,1 ans avec des extrêmes de 22 à 56 ans. La prévalence du tabagisme était de 16,3% (n=62) de la population d'étude dont 32,7% (n=52) chez les hommes contre 4,5 % (n=10) chez les femmes. L'âge moyen de début du tabagisme était de 19 ans avec des extrêmes de 11 à 29 ans avec une consommation moyenne de 9 cigarettes/jour. 13% (n=50) des personnes fumaient aussi le narguilé, 9% (n=34) consommaient de l'alcool et 3% (n=21) des personnes consommaient du Cannabis. 67,7% des fumeurs (n=42) se projetaient d'arrêter de fumer, dont 30,9% (n=13) dans les 3 prochains mois, 52,4% (n=22) dans les prochains 6mois et 16,7% (n=16) se projetaient d'arrêter dans l'année. Plusieurs activités incitaient les fumeurs à fumer davantage dont la garde, la pause-café, et les repas dans respectivement 90,3% (n=56), 64,3% (n=40) et 61,3% (n=38) des cas. A la suite de cette enquête, une réflexion doit être menée sur la nécessité de mener des actions de sensibilisation pour renforcer la motivation de ceux qui désirent arrêter et les aider dans leur démarche de sevrage.

## Abstract (en)

Smoking is a major public health problem. Doctors and paramedical staff are not excluded from this plague. Smoking ban in hospitals originated from government effort to reduce passive smoking. The objectives were to evaluate smoking habits among doctors and paramedical staff in order to implement tobacco control strategy in this study population and to refer them to the smoking-cessation counselling. We conducted a descriptive cross-sectional study of the entire staff of the Hospital University Center Mohammed VI, Marrakech based on the distribution of anonymous questionnaires. A total of 530 questionnaires were distributed, and 380 were returned, a response rate of 71.7%. The study population consisted of 58.2% women (n=221) and 41.8% men (n=159). Doctors (n=220) were the most represented occupational category (57.9%) followed by nurses (31.8%). Smokers (n=62) accounted for 16.3% of our study population; the ex-smokers (n=31) accounted for 8.1% and the non-smokers (n=287) 75.5%. The average age of smokers was 31.1 years, ranging from 22 to 56 years. The prevalence of smoking was 16.3% (n=62) of study population, of whom 32.7% (n=52) among men compared to 4.5% (n=10) among women. The average age of smoking onset was 19 years with a range from 11 to 29 years and with a mean consumption of 9 cigarettes/day. 13% (n=50) of people even smoked narguilé, 9% (n=34) consumed alcohol, and 3% (n=21) cannabis. 67.7% of smokers (n=42) were planning to quit, of whom 30.9% (n=13) in the next 3 months, 52.4% (n=22) in the next 6 months and 16, 7% (n=16) were planning to quit in the year. Several activities encouraged smoking, including night shift, coffee breaks and meals in 90.3% (n=56), 64.3% (n=40) and 61.3% (n=38) of cases respectively. This survey highlights the need to carry out awareness-raising actions to strengthen people motivation to quit smoking and help them during their withdrawal.

## Introduction

Le tabagisme est un problème majeur de santé publique. Le tabac, première cause évitable de mortalité, tue chaque année 5 millions de personnes dans le monde et 73 000 en France. En effet, il est cité comme un facteur étiologique de la presque totalité des affections de tous les appareils ou systèmes surtout respiratoire, cardiovasculaire, digestif et génito-urinaire [1]. Malgré la connaissance de la morbi-mortalité liée au tabagisme et les efforts déployés pour son éradication, le tabac continue à faire des ravages et à conquérir de nouveaux adeptes. La prévalence mondiale de fumeurs est estimée par l'OMS, en 2005, à 1,3 milliards dont 800 millions dans les pays en développement [2] En France, l'année 2003 a été marquée par une diminution importante (24%) de la prévalence du tabagisme du personnel hospitalier. Cette diminution étant retrouvée à la fois chez les fumeurs quotidiens (au moins une cigarette par jour) et chez les fumeurs occasionnels (moins d'une cigarette par jour). Vingt-quatre pour cent des personnes se déclaraient ex-fumeurs et 52% comme n'ayant jamais fumé. Au Maroc, de nombreux efforts sont déjà faits dans le domaine de la prévention du tabagisme. L'interdiction de fumer à l'hôpital est née d'une volonté gouvernementale de réduire le tabagisme passif. Le personnel soignant n'est certes pas épargné par ce fléau. Une étude réalisée à Casablanca en 2002 avait montré que la prévalence globale du tabagisme était de 14,9%, variant de 12,5% chez les paramédicaux, à 15,5% chez les agents de service, à 16,2% chez les médecins, à 17,1% chez les biologistes, et à 22,2% chez les administratifs [3].

## Méthodes

Il s'agit d'une étude transversale descriptive s'étalant sur une durée de 3 mois entre février et avril 2016 portant sur l'ensemble du personnel soignant médical et paramédical du CHU de Marrakech. L'enquête s'est faite en se basant sur la distribution des questionnaires anonymes pour l'ensemble du personnel soignant au sein des différents services du CHU, qui ont été recueillis et analysés de façon anonyme. Le respect de l'anonymat ainsi que la confidentialité ont été pris en considération lors de la collecte des données.

## Résultats

**Figure 1:**
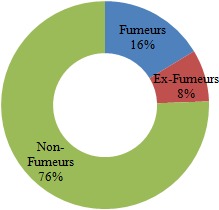
Taux du tabagisme au sein du personnel

**Figure 2:**
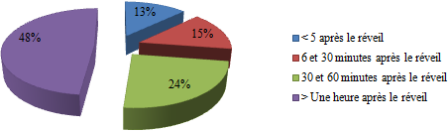
Délai entre réveil et la 1^ère^ cigarette fumée

**Figure 3:**
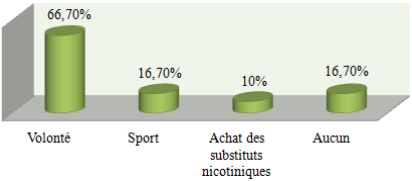
Moyens d’aide au sevrage

**Figure 4:**
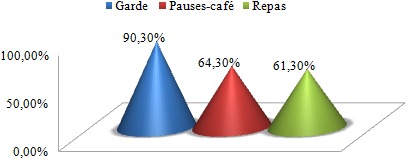
Les activités incitant le fumeur à fumer

**Figure 5:**
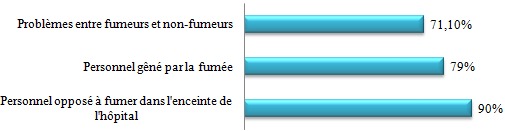
Personnel et tabagisme au sein de l’hôpital

Nous avons distribué 530 questionnaires au personnel médical et paramédical du CHU Mohamed VI dont 380 questionnaires ont été récupérés, soit un taux de réponse de 71,69%. La population d'étude était constituée de 58,2% de femmes (n= 221) et de 41,8% d´hommes (n=159). Le personnel médical (n=220) était la catégorie professionnelle la plus représentée (57,9%) suivie des infirmiers (31,8%) (Tableau 1). 53,1% (n=202) de l'ensemble du personnel avait des horaires fixes alors que 46,9% (n=178) avait des horaires variables. 18,4% des personnes interrogés (n=70) ne travaillaient jamais la nuit, 77,6% travaillaient des fois la nuit alors que 4% du personnel ne travaillaient que la nuit. La moyenne d'âge de notre population était de 33,9 ans, avec des extrêmes de 22 et de 59 ans. Elle était de 32,6 ans chez les hommes et de 34,9 ans chez les femmes. Les fumeurs (n = 62) représentaient 16,3% de notre population d'étude; les ex-fumeurs (n=31) 8,1 % et les non-fumeurs (n= 287) 75,5% (Figure 1). 13% (n=50) des personnes fumées le narguilé, 9% (n=34) consommaient de l'alcool et 3% (n=21) des personnes consommaient du Cannabis. La moyenne d'âge des fumeurs était de 31,1 ans avec des extrêmes de 22 à 56 ans. La prévalence du tabagisme est de 16,3% (n=62) de la population d'étude dont 32,7 % (n=52) chez les hommes contre 4,5 % (n=10) chez les femmes. L'âge moyen de début du tabagisme était de 19 ans avec des extrêmes de 11 à 29 ans avec une consommation moyenne de 9 cigarettes/jour. 13% des patients fument leur première cigarette < 5 minutes après le réveil, 15% des patients entre 6 et 30 minutes, 24% entre 30 et 60 minutes et 48,4% des patients fument leur première cigarette plus d'une heure après le réveil (Figure 2). 67,7% des fumeurs (n=42) se projettent d'arrêter de fumer, dont 30,9% (n=13) dans les 3 prochains mois, 52,4% (n=22) dans les prochains 6mois et 16,7% (n=16) se projettent d'arrêter dans l'année. Parmi les fumeurs qui ont entrepris une tentative d'arrêt de tabac (plus de 7 jours) ces 12 derniers mois, la majorité n'ont pas fait appel aux structures d'aide au sevrage de notre hôpital (93,3% des cas), par contre ils ont fait appel à la volonté et au sport dans respectivement 66,6% et 16,7% des cas. Seuls 10% des cas ont été tentés par l'achat des substituts nicotiniques, par contre 16,7% de ses personnes n'ont fait appel à aucun moyen de sevrage dans leur tentative d'arrêt du tabac (Figure 3). Parmi les fumeurs, 54,8% des cas considéraient qu'ils pouvaient arrêter de fumer facilement s'ils le voulaient. 67,7% des fumeurs veulent arrêter (n=42) pour plusieurs raisons, principalement pour préserver leur santé ou pour des raisons économiques. Plusieurs activités incitaient les fumeurs à fumer davantage dont la garde, les pauses-café, et les repas dans respectivement 90,3% (n=56), 64,3% (n=40) et 61,3% (n=38) des cas (Figure 4). 2 personnes (3,2% de l'ensemble des fumeurs) ont reconnu avoir déjà fumé en pleine réunion. 31 sujets (13,4%) déclaraient être des ex-fumeurs. Ils avaient en moyenne arrêté de fumer à l'âge de 27 ans avec des extrêmes de 17 à 49 ans. En moyenne, ils fumaient 8 cigarettes par jour. Sur l'ensemble du personnel médical et paramédical du CHU Mohamed VI, 79% des cas (n=300) affirmaient être régulièrement gênés par la fumée des autres personnes au travail, 90% des cas (n=342) s'opposaient au fait de fumer dans l'enceinte de l'hôpital (en dehors des lieux réservés) et 71,1% des cas (n=270) avouaient l'existence des problèmes entre fumeurs et non-fumeurs (Figure 5). 84,2% (n= 320) du personnel du CHU n'étaient pas au courant de l'existence d'une circulaire ministérielle interdisant de fumer dans les établissements de santé. 95,2% (n=362) de ces derniers étaient parfaitement au courant des méfaits du tabac, mais seul 39,7% abordaient le problème du tabac avec leurs patients.

**Tableau 1:** Répartition de l’ensemble du personnel sujet de l’enquête

	Nombre	Pourcentage
Médecins	220	57,9 %
Infirmiers	120	31,8 %
Techniciens	8	2,1 %
Sage femme	2	0,5 %
Autres	30	7,9 %

## Discussion

**Figure 6:**
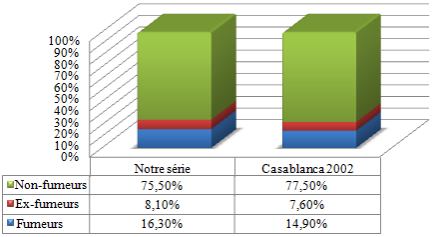
Comparatif du taux de tabagisme entre notre étude et une étude à Casablanca en 2002

Le tabagisme au sein des centres hospitaliers est un véritable problème de santé, qui a fait l'objet de plusieurs études. L´hôpital doit être un lieu sans tabac afin que son personnel puisse servir de modèle pour les patients, car le tabagisme altère l´image présentée au public par un personnel médical et paramédical supposé servir d'exemple pour contribuer de façon optimale à la lutte antitabac vu la liaison étroite entre le tabagisme du personnel hospitalier et son attitude par rapport à la lutte antitabac. La prévalence globale du tabagisme chez le personnel médical et paramédical varie en fonction des études entre 10% et 51,7% [4, 5]. Elle dépend de plusieurs facteurs, dont l'âge, le sexe, la profession et la région. Les fumeurs (n = 62) représentaient 16,3% de notre population d'étude, les ex-fumeurs (n=31) 8,1% alors que les non-fumeurs représentaient la grande majorité avec 75,5% de l'ensemble du personnel. En 2002, une étude nationale au Maroc avait trouvé à Casablanca des chiffres similaires à notre étude avec près de 14,9% de fumeurs, 7,6% d'ex fumeurs et 77,5% de non-fumeurs [3] (Figure 6). Une méta-analyse de 7 études sur le tabagisme chez le personnel de santé [6–12] réalisées au Maroc de 1990 à 1997 a trouvé une prévalence globale de tabagisme élevée, estimée à 38,2%, ce qui montre la nette diminution du taux global des fumeurs durant ses dernières années (abstraction faite du sexe). Une méta-analyse dans 45 hôpitaux catalans a trouvé une prévalence globale du tabagisme de 28,1 % avec des extrêmes allant de 40,3% et 19,1% [13]. L'enquête Baromètre personnel hospitalier [14] réalisée entre 2002 et 2003 au niveau de 58 établissements répartis sur l'ensemble du territoire français avait montré une prévalence du tabagisme du personnel hospitalier de 31% en 2002, suivie d'une baisse importante en 2003 avec une prévalence estimée à 24%. Selon le genre, La prévalence du tabagisme est de 32,7% (n=52) chez les hommes contre 4,5% (n=10) chez les femmes, soit un sexe ratio de 6,2. On retient donc la nette prévalence du tabagisme chez les hommes par rapport aux femmes dans notre contexte. Malgré une augmentation progressive ces dernières années. Cette prédominance masculine peut être expliquée dans les pays arabo-musulmans par des raisons socio-culturelles, où la femme fumeuse est très mal vue. Quoique cette disparité homme-femme en matière de tabagisme a tendance à diminuer dernièrement. Alors qu'à l'échelle internationale [13–15], la différence entre les deux sexes est moins importante voire même plus élevée chez les femmes dans certains pays. Une donnée également retrouvée dans l'enquête « Baromètre tabac personnel hospitalier 2003 » où 23% des femmes fument contre 26% des hommes. Dans notre étude la moyenne d'âge des fumeurs était de 31,1 ans avec des extrêmes oscillant entre 22 et 56 ans. L'âge moyen du personnel fumeur est variable selon la région et l'ancienneté d'enquête : elle varie entre 21 et 32 ans [5, 13, 16]. En Espagne [17], 23,6% des médecins fumeurs sont âgés de moins de 30 ans. En Algérie, Liban, Egypte [18, 19], 45% des fumeurs de sexe masculin sont âgés entre 25 et 60 ans. L´âge moyen de début du tabagisme est relativement précoce. Dans notre étude, il était de 19 ans avec des extrêmes allant de 11 à 29 ans. Les motivations du tabagisme sont dominées par les gardes, les pauses-café, et les repas dans respectivement 90,3% (n=56), 64,3% (n=40) et 61,3% (n=38) des cas, alors que dans d'autres travaux c'est le plaisir qui motivait le plus à fumer [20–22], ou alors le suivisme qui était le plus souvent rapporté dans une méta-analyse. Les raisons fortes d´un éventuel arrêt du tabagisme sont intéressantes à connaître. Dans notre travail 67,7% des fumeurs veulent arrêter (n=42) pour plusieurs raisons, principalement pour préserver leur santé ou pour des raisons économiques. Dans certaines études [6–12] les principales raisons d'arrêter de fumer étaient : donner le bon exemple à ses enfants ou aux enfants en général, promouvoir la santé et la discipline personnelle. Il est remarquable que l´économie d´argent vienne souvent en dernier lieu. Le taux de fumeurs sur le lieu du travail avant l´apparition de la loi antitabac était de 54,6%. Dans notre étude une légère baisse a donc été enregistrée, malgré que 84,2% (n= 320) du personnel de notre travail n'étaient pas au courant de l'existence d'une circulaire ministérielle interdisant de fumer dans les établissements de santé. Nous signalons que la loi marocaine antitabac interdit de fumer dans tous les lieux destinés à un usage collectif notamment dans les hôpitaux, cliniques, maisons de convalescence, centres de santé et services de prévention de toute catégorie. Selon les études, le taux de personnels de santé qui déclarent fumer devant les patients varie entre 11% et 52% en Europe, les taux les plus souvent cités étant de 30% à 40% [23]. A la lumière de ces résultats, nous constatons que la prévalence du tabagisme sur les lieux de travail à l'échelle nationale et internationale reste très élevée malgré l'interdiction légale de fumer à l'hôpital dans ces pays. Dans notre étude, seuls 39,7% du personnel abordaient le problème du tabac avec leurs patients malgré que 95,2% (n=362) de ces derniers étaient parfaitement au courant des méfaits du tabac. Des taux plus élevés sont notés dans des études nationales [24, 25]

## Conclusion

Ce travail nous a permis d'apprécier la prévalence du tabagisme de l'ensemble du personnel médical et paramédical du CHU Mohammed VI de Marrakech. À la suite de cette enquête, une réflexion doit être menée sur le type d'action à réaliser pour renforcer la motivation de ceux qui désirent arrêter et les aider dans leur démarche de sevrage. Cette dernière va servir de pierre angulaire pour orienter la lutte antitabac. Les pouvoirs publics devraient aussi intensifier la sensibilisation de la population et particulièrement le personnel hospitalier afin que l´hôpital devienne un lieu sans tabac et que son personnel puisse servir de modèle pour les patients.

### Etat des connaissances actuelle sur le sujet

- Le tabagisme constitue un enjeu majeur en termes de santé publique dans le monde entier, en raison de la mortalité et de la morbidité qui lui sont liées. Il constitue la première cause de décès évitable dans le monde;
- La plupart des enquêtes actuelles mettait en évidence un niveau de tabagisme comparable entre les professionnels de santé et la population générale;
- Le personnel hospitalier a un rôle à jouer dans la lutte contre le tabagisme de par son exemplarité et dans l'aide au sevrage tabagique.

### Contribution de notre étude à la connaissance

- La prévalence du tabagisme parmi le personnel de santé hospitalier au Maroc n'est pas connue alors que le tabagisme est en expansion au Maroc, en Afrique et dans le reste du monde selon l'OMS;
- Faire un état des lieux du tabagisme au sein du personnel médical et paramédical du CHU de Marrakech;
- Cerner les populations exposées et ouvrir la voie à des actions de prévention ciblées dont on peut attendre un meilleur impact.
